# Everybody's Page

**Published:** 1890-12-20

**Authors:** 


### EVERYBODY'S PAGE.

A PIERCING shriek brought the agitated mother hurriedly from the adjoining room. "What is it, my daughter?" she excitedly exclaimed. "Has this person from New York been uncivil?" "Not intentionally, mother," faintly replied the fair young Bostonian. "But it was a painful shock. He said 'hippopotamuses,' instead of 'hippopotami.'"

THE medical properties of turpentine as well as its cleansing ones are pretty generally known to housekeepers. Those unfortunate people who are now being unpleasantly reminded of the presence of winter by the excruciating torture of their chilblains will not be slow to recognise its value. As a preventive against moths and other more unwelcome visitors its value is not so widely known. A few drops poured now and again in cupboards and store-rooms will be found effectual, and we doubt if any furniture which is regularly cleaned with turpentine is ever visited by "the terror by day."

THERE are those in the world who are in doubt still as to M. Pasteur's treatment for the bite of a mad dog; here is an old prescription, nice and mild, which may, we think, be safely described as not likely to do much harm. "For the bite of a mad dog take two quarts of strong ale, two pennorths of treacle, two garlic heads, a handful of cinquefoil, sage, and rue; boil all together to a quart, strain it, and give to the patient three or four times a day." Then for the treatment of the wound: "Take dittany, agrimony, and rusty bacon beaten well together, and apply to the sore, to keep it from festering."

THAT there are not more outbreaks of typhoid fever in French towns, such as that in the garrison at Caen which has just been made the subject of a report by the Académie de Médecine at Paris, is apparently a matter of good luck rather than good management. Seeing that a municipality in France has no power to prevent the use of water contaminated by sewage, no bill of mortality, however high, should be a cause of surprise. If, as seems probable, the outbreak at Caen will lead to the investing of municipalities with the necessary powers, it will be an instance of good coming out of evil.

THE *Magazine of American History* states that while in command at Rock Island during the Blackhawk war, General Henry Dodge's forces were attacked by cholera, which was to a great extent induced by the intemperate habits of the troops. The General's order book contains the following peremptory order, issued with a view to checking the excess: "Every soldier or ranger who shall be found drunk or sensibly intoxicated shall be compelled to dig a grave at a suitable burial-place large enough for his own reception, as such grave cannot fail soon to be wanted for the drunken man or some drunken companion." History does not say whether the drunken soldier was to be compelled to dig his grave while still inebriated. But the General, apparently, had not so much to complain of if his soldiers were only *sensibly* intoxicated; it has been our invariable misfortune when coming across a drunkard to find him the reverse of sensible.

THE indiscriminate use of dried mushrooms in soups and stews on the continent has led to unfortunate results in many cases, especially, it would seem, in Berlin, where the police are stated to have issued a caution against their consumption. The assertion that poisonous fungi are sometimes dried with edible mushrooms is sufficiently probable to cause no surprise.

THE discoveries of Dr. Perlewitz and Dr. Hann as to the influence of the towns of Berlin and Vienna respectively upon their climate are remarkable rather for their simplicity than originality. That a town is always warmer than the open country outside, and that the greatest daily differences of temperature are to be found in the evening, owing to a retardation of radiation in the town, are self-evident propositions which scarcely require the intellect of a *savant* for their discovery.

THE thirteen rules for the maintenance of health which have just been circulated in all the military stations in India, by order of the Commander-in-Chief, are so generally applicable as to be worthy of circulation in every home in England The warnings that less meat and more fruit and vegetables would be more conducive to health, that spirits should be eschewed, that sleep should be taken before, not after meals, that the value of wearing flannel even in hot weather cannot be estimated too highly, are common-sense ones, which many people in England, as well as in India, might take to heart with advantage.

THE manager of the London Road Car Company (Limited) writes, under date 9th inst.: "In the *Evening Standard* of Saturday last a paragraph appeared, which was alleged to have been copied from THE HOSPITAL, stating that this company lost seventeen horses in six days from a disease known as 'pink eye.' As the statement is utterly untrue, and calculated to do this company harm, I must ask you to give insertion to this contradiction in your first issue." We are very glad to insert this contradiction, and to hear on the manager's authority that there is no foundation for the statement in question.

MODELLED on the District Telegraph System of the United States which has been in successful operation for some time past, a somewhat similar system has now been organized in London under the name of the District Messenger Service and News Company. The head office is in Lime Street in the City and there are already four district offices in the West-End. Any householder in any of the districts can have a call box fixed in his house free of charge by which he can signal to the district office for a messenger, a cab, police, or firemen. These call-boxes should prove of great use to the householder in case of burglary, and to many institutions where, when the secretary puts on his hat and walks out, the the whole staff goes with him. The company will be responsible for the good behaviour and faithful performance of the duties of their servants to the extent of £20.

